# Comparative Retinal Morphology of Two Sympatric Lizard Species from Distinct Microhabitats

**DOI:** 10.3390/ani16121799

**Published:** 2026-06-10

**Authors:** Yan-Ting Fu, Wei-Zhen Gao, Lei Shi

**Affiliations:** 1 Xinjiang Key Laboratory for Ecological Adaptation and Evolution of Extreme Environment Organism, College of Life Sciences, Xinjiang Agricultural University, Urumqi 830052, China; yantingfu011@163.com (Y.-T.F.); gao7222@yeah.net (W.-Z.G.); 2 College of Animal Sciences, Xinjiang Agricultural University, Urumqi 830052, China

**Keywords:** lizards, retina, photoreceptors, ultrastructure, ecological adaptation

## Abstract

This study investigated retinal interspecific differences between two sympatric lizards from the Turpan Basin of Xinjiang that occupy distinct microhabitats. *Eremias roborowskii* inhabits shrublands, while *Phrynocephalus axillaris* lives in open sandy areas. We adopted retinal whole-mounting, paraffin sectioning, scanning electron microscopy (SEM), and transmission electron microscopy (TEM) to compare retinal structure, oil droplet traits, photoreceptor arrangement, and ultrastructural characteristics between the two species. Both species exhibited five types of colored oil droplets, a temporal fovea, and a highly vascularized conus papillaris, confirming cone-dominant retinas. *P. axillaris* had larger oil droplets with an average diameter of 3.719 ± 1.100 μm. In contrast, *E. roborowskii* showed a higher oil droplet density of 1473 ± 1203 ind/mm^2^. The inner plexiform layer (IPL) was significantly thicker in *E. roborowskii* (56.01 ± 14.76 μm) relative to *P. axillaris* (50.76 ± 15.25 μm). The two species also differed markedly in photoreceptor ultrastructure. *E. roborowskii* contained mitochondria-rich ellipsoids, while *P. axillaris* possessed glycogen-enriched paraboloids. These structural variations reflect compatibility with local ambient light conditions. *E. roborowskii* is predisposed to suit diffuse shrubland light through high droplet density and mitochondrial-dominant ellipsoids, whereas *P. axillaris* tends to accommodate intense open-field sunlight via larger oil droplets and well-developed paraboloids. This study provides key morphological evidence to reveal how microhabitat divergence drives retinal specialization among desert reptiles.

## Simple Summary

This research focused on two sympatric lizard species that coexist in the Turpan Basin of Xinjiang, each adapted to divergent microhabitats: *Eremias roborowskii* thrives in shrublands with scattered light, while *Phrynocephalus axillaris* occupies open, barren sandy regions exposed to intense, direct solar radiation. We utilized a combination of light and electron microscopic methodologies to analyze their retinal anatomy. Our findings indicated that both species possess five distinct types of pigmented oil droplets, which function as spectral filters to enhance color perception. Notably, *P. axillaris* exhibited larger oil droplet diameters, whereas a higher overall droplet density was observed in *E. roborowskii*. Additionally, *E. roborowskii* featured a thicker inner retinal layer, potentially improving its ability to detect moving prey in intricate ambient lighting, whereas *P. axillaris* displayed more advanced cellular structures adapted to mitigating retinal phototoxicity under intense surface glare.

## 1. Introduction

**Figure 1:**
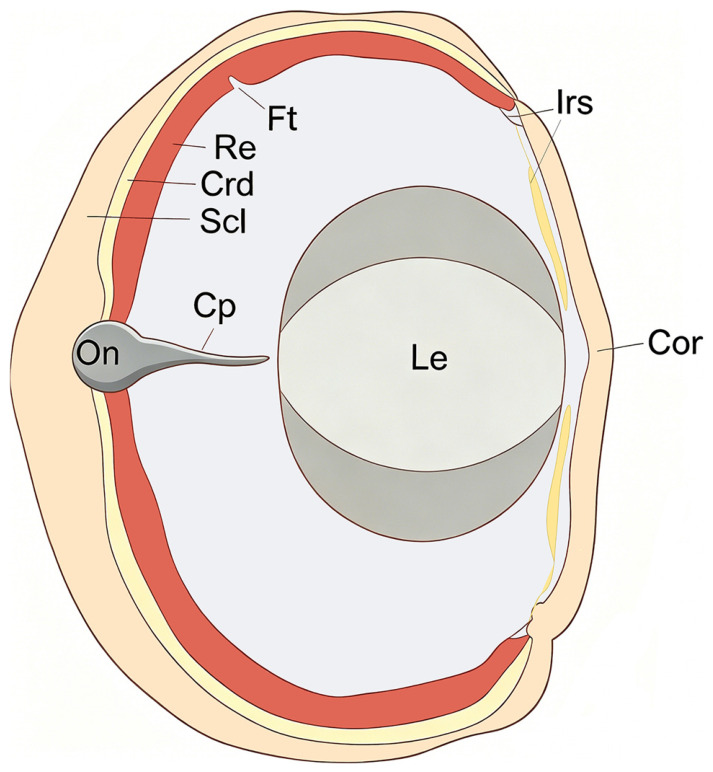
Schematic diagram of the typical ocular structure in lizards [8]. **Abbreviations:** Cor—cornea; Irs—iris; Crd—choroid; Scl—sclera; On—optic nerve; Cp—conus papillaris; Re—retina; Le—lens; Ft—temporal fovea.

Light plays an essential role in the ecology of all living organisms, significantly influencing their survival and creating strong selective pressures that have shaped the evolution of the vertebrate eye, an intricate sensory structure (Figure 1) [1]. The primary role of the eye is to gather and process visual information, with evolutionary modifications in retinal architecture, cellular makeup, and detailed morphology directly facilitating adaptations to species-specific photic environments [2]. Vertebrate retinas contain two distinct types of photoreceptors: rods and cones. Although most vertebrates have both types, their relative abundance varies significantly based on the ecological behaviors and light environments of different species [3]. Species that are active at night typically exhibit retinas dominated by rods, whereas those active during the day usually have a higher concentration of cones [4,5,6,7].

The retina of a daytime-active lizard is predominantly composed of cones and is frequently associated with features like a fovea and oil droplets [9,10,11]. Retinal whole-mount preparations were utilized to investigate the distribution of these oil droplets and their role in light adaptation [12]. Scanning electron microscopy (SEM) has revealed that the retina of the ribbon snake (*Thamnophis proximus*) comprises four distinct types of cones, and initial studies have elucidated how photoreceptor topography correlates with ambient illumination [13]. Additionally, transmission electron microscopy (TEM) has confirmed that the photoreceptor layer consists exclusively of single and double cones in the retina of *Trachylepis quinquetaeniata* [14]. In diurnal lizards, the outer segments of the cones are usually conical, while the inner segments feature an ellipsoid rich in mitochondria and a paraboloid abundant in glycogen, structural adaptations that act as waveguides to maximize photon capture by the photopigments within the outer segment [14,15]. Importantly, the extent of development of the ellipsoid and paraboloid exhibits remarkable interspecific variation; for instance, in *T. quinquetaeniata*, the mitochondria in the ellipsoid are distributed in a gradient, with larger ones centrally located and smaller ones at the edges, while in some sand-dwelling skinks (*Scincus scincus* and *Eumeces schneideri*), the glycogen accumulation in the paraboloid is more significant [1,14].

Oil droplets are distinctive carotenoid-rich structures found in the inner segments of avian and reptilian cones. Positioned at the apex of the ellipsoid, these droplets allow light to filter through before it is absorbed by the visual pigments located in the outer segment [16,17]. The presence of colored oil droplets cuts off short-wavelength light entering the outer segments, which in turn alters spectral sensitivity and plays a role in enhancing color contrast and reducing chromatic aberration [17]. Research on retinal wholemounts indicates that *Anolis* lizards have developed various types of oil droplets, such as green, yellow, and clear forms, to align with the absorption peaks of different visual pigments, thereby creating a specialized system for color vision [18]. Additionally, certain skinks that live in sandy or burrowing habitats, where light is limited, have adapted unique visual characteristics that enable them to thrive under fossorial low-light conditions as well as intense surface glare [1,14].

**Figure 2:**
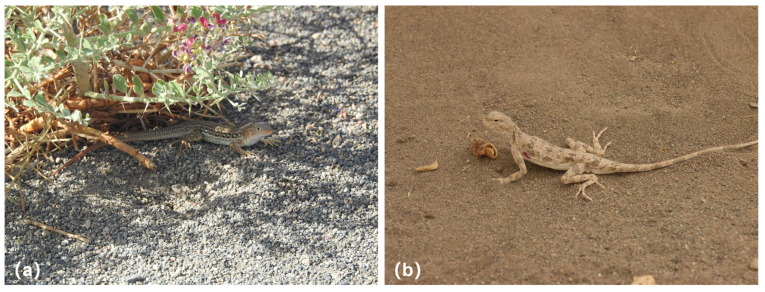
Habitat of *Eremias roborowskii* (**a**) and *Phrynocephalus axillaris* (**b**).

Sympatric species frequently attain coexistence and resource division via niche differentiation. Variations in microhabitat characteristics, lighting conditions, and foraging strategies often lead to coordinated adaptive changes in visual structures, among which the genus *Anolis* has been most intensively studied [1,3,18,19,20,21]. Nevertheless, there is a lack of comparative research exploring the connections between retinal microstructure, ultrastructural traits, and microhabitats among sympatric lizards. *Eremias roborowskii* and *Phrynocephalus axillaris* are two lizard species that coexist in the Turpan Desert of Xinjiang, displaying significant differences in their microhabitat preferences: *E. roborowskii* primarily resides in low fine-sand dunes with shrub coverage (Figure 2a) [22], while *P. axillaris* is mostly located in open, sandy regions (Figure 2b) [23]. Foraging behavior plays a crucial role in the evolutionary adaptations of lizards [24,25,26,27], which can be classified into two main foraging strategies: sit-and-wait (SW) and active foraging (AF). *E. roborowskii* employs an AF approach to hunt for prey hidden among shrubs, whereas *P. axillaris* typically uses a SW strategy, ambushing agile prey with quick, short-distance movements (unpublished data).

Considering these elements, we chose two coexisting lizard species that exhibit different preferences for their microhabitats. Our goal is to employ an integrative methodology encompassing histomorphometry, scanning electron microscopy (SEM), and transmission electron microscopy (TEM) to analyze and contrast the retinal structures of these two daytime-active lizard species, thereby elucidating the adaptive features of their retinas that have developed in relation to their microhabitat environments throughout evolution.

## 2. Materials and Methods

### 2.1. Experimental Animals

Lizard specimens of *Eremias roborowskii* (*n* = 12) and *Phrynocephalus axillaris* (*n* = 9) were collected from the Turpan Basin (88°45′ E, 42°49′ N, Xinjiang Uygur Autonomous Region) from May 2024 to September 2025. All sampled individuals were healthy adults. Animals were allocated to different experimental groups as follows: three individuals per species were used for oil droplet quantification (*n* = 3); eight *E. roborowskii* and four *P. axillaris* were processed for histological sectioning; another three individuals of each species were used for scanning electron microscopy (SEM) (Xinjiang Institute of Ecology and Geography, Chinese Academy of Sciences, Urumqi, China) and transmission electron microscopy (TEM) (Wuhan Servicebio Technology Co., Ltd., Wuhan, China), with left and right eyes employed independently for the two techniques.

Each lizard was housed individually in a 120 × 60 × 60 cm^3^ cage lined with 5–10 cm fine sand substrate. A heat lamp (Foshan Electrical and Lighting Co., Ltd., Foshan, China) was fitted to maintain thermal and photoperiod conditions under a 12 h light:12 h dark cycle. Overnight temperature was maintained at 22 °C (19:00–07:00), and daytime temperature ranged from 25 to 30 °C (07:00–19:00). During the 7-day acclimation period, lizards were fed live *Tenebrio molitor* larvae three to five times weekly with ad libitum fresh water supply [1].

All animal experimental protocols were approved by the Animal Welfare and Ethics Committee of Xinjiang Agricultural University (Approval No. 2023014).

### 2.2. Sample Preparation

#### 2.2.1. Animal Euthanasia and Eye Enucleation

Tissue samples were collected in alignment with the daily activity patterns of the species under investigation. Individuals of *E. roborowskii* and *P. axillaris* were euthanized during the daytime [10,28]. Before obtaining tissue, the lizards were sedated through an intraperitoneal injection of tricaine methanesulfonate (MS222; 250 mg/kg) (Sigma-Aldrich, St. Louis, MO, USA) [8]. Once they exhibited total unconsciousness and a lack of withdrawal reflexes, a fatal dose of MS222 was given through an intracardiac injection, followed by cervical dislocation to confirm effective euthanasia.

All ocular surgical procedures were performed within a sterile, laminar flow environment. Following the previous step of eyeball isolation, an incision around the orbit was created with ophthalmic scissors, followed by careful dissection and separation of the extraocular muscles and adjacent connective tissues using forceps. The removed eyeballs were promptly placed in appropriate fixatives designed for future histological and ultrastructural examinations [8].

#### 2.2.2. Retinal Whole-Mount Preparation

For retinal whole-mount preparation (*n* = 3 for each species), the method described by Campbell et al. was followed [12]. Enucleated eyes were immersed in 0.01 M PBS. A micro-incision was made near the corneal limbus to identify the dorsal orientation, after which PBS was gently injected into the posterior chamber. The samples were then incubated at 4 °C overnight (up to 14 h) to facilitate the mechanical separation of the neural retina from the underlying retinal pigment epithelium (RPE). The next day, corneal scissors were utilized to excise the cornea and remove the lens and vitreous body.

Once the retina was separated, small radial incisions were made along its perimeter to flatten it. The retina was then cut into irregular shapes to identify dorsal, ventral, nasal, and temporal orientations. Blunt needles were used to excise the central papillary process of the optic disc, leaving a hole as a reference point. These retinal landmarks helped confirm orientation and identify specific regions of interest. Small sections of retinal tissue were affixed to 24 × 52 mm^2^ adhesive slides (Jiangsu Citotest Labware Manufacturing Co., Ltd., Nantong, China) with the photoreceptor side facing up. A drop of PBS was added to maintain tension in the retina, and a coverslip was placed on top to create retinal flat mounts [12]. Fine brush, dipped in a small quantity of gum arabic, was used to seal the edges of the coverslip [29], allowing the retina to remain in a temporary state for up to 48 h for counting. The samples were examined under a Motic conventional light microscope (Motic China Group Co., Ltd., Xiamen, China) at 40× magnification with tungsten lamp illumination.

#### 2.2.3. Tissue Section Preparation and Observation

For histological sections, right after the removal of the eyeballs, each intact eyeball was placed in 20 mL of FAS eye fixative, maintaining a tissue-to-fixative ratio of 1:20, and carefully held with forceps (*E. roborowskii n* = 8, *P. axillaris n* = 4). FAS eye fixative is prepared by mixing glacial acetic acid, formaldehyde, absolute ethanol and normal saline by volume; it is specially formulated for rapid fixation of ocular tissues to effectively prevent retinal detachment and tissue shrinkage [12]. A 0.01 mm disposable needle was used to puncture the cornea from the top. The needle was slightly pulled back into the eyeball to inject a small amount of fixative. To ensure proper orientation for later sectioning, the limbus was tied off with a sterile surgical suture at the 12 o’clock position (dorsal side). After being fixed at room temperature for 24 h, the FAS fixative was replaced with 4% PFA for an additional 48 h. Following this, the 4% PFA was discarded, and the sample was washed with PBS buffer for one hour. The sample then underwent dehydration through a series of ethanol solutions (50%, 70%, 80%, 90%, 95%, and 100%, each for one hour, repeated twice). After dehydration, the eyes were immersed in xylene three times for 20 min each, followed by three changes of paraffin at 65 °C for two hours each. The eyes were embedded in paraffin with longitudinal sections, and serial sections of 5–8 μm thickness were cut horizontally. These sections were placed on adhesive slides, baked at 50 °C for two hours, stained according to standard hematoxylin and eosin (H&E) methods, and then mounted with neutral resin before the xylene evaporated [30]. Complete serial sections were examined and photographed using a light microscope with Motic Images Plus 3.1 (x64) software.

#### 2.2.4. SEM Sample Preparation and Observation

For TEM sample preparation, the opposite eye was utilized for SEM analysis (*E. roborowskii* and *P. axillaris*: *n* = 3 for each species). After excising the cornea, lens, and other eye tissues, the retinas were dissected in a 2.5% glutaraldehyde solution and fixed at ambient temperature for 24 h. Following this, they were rinsed with a phosphate buffer (0.1 M, pH 7.8) and subsequently post-fixed with 1.0% osmium tetroxide at room temperature for one hour. The retina underwent dehydration through a series of ethanol solutions with increasing concentrations, then infiltrated with hexamethyldisilazane (HMDS) and allowed to evaporate overnight. The retina was then attached to a metal stub with the photoreceptor side facing outwards and coated with gold–palladium using a Hitachi E-1045 sputter coater (Hitachi High-Technologies Corporation, Tokyo, Japan) under the following parameters: 5 mA current, 120 s coating time, resulting in a 10–15 nm thick conductive film. Observations and image capture were carried out with a Thermo Scientific Helios 5 CX (Thermo Fisher Scientific, Inc., Waltham, MA, USA) scanning electron microscope set at an accelerating voltage of 2.0 kV and a working distance of 6.4–6.8 mm, and images were acquired at magnifications ranging from 500× to 1000× [13].

#### 2.2.5. TEM Sample Preparation and Observation

To conduct an ultrastructural study of the retina, three samples from each species were chosen (*E. roborowskii* and *P. axillaris*: *n* = 3 for each species). This method was adapted from the procedures outlined in [31].

Once the eyes from both species were extracted, the cornea, iris, lens, and vitreous body were swiftly removed, followed by the dissection and fixation of the central retina. This entire procedure was executed promptly: the tissues were immersed in a chilled 2.5% glutaraldehyde solution right after being taken from the living organism [17,28]. After a fixation duration of 24 h, the samples underwent rinsing in 0.1 M PBS and were subsequently post-fixed in 2% osmium tetroxide for a period of 2 h. Post the initial fixation, the samples were washed with chilled 0.1 M phosphate buffer (at 4 °C) every 15 min for 2 h. Dehydration was achieved through a series of ethanol solutions of increasing concentration. Infiltration was gradually conducted using a mix of dehydrating and embedding agents, culminating in embedding with Epon resin. The eye cups were meticulously positioned before polymerization to facilitate the collection of longitudinal or tangential sections through the fovea [14].

The specimens were positioned on the holder of an ultramicrotome that featured a diamond blade, allowing for the cutting of serial sections measuring 50–70 nm in thickness. These sections were then transferred onto 150-mesh copper grids that had been coated with Formvar film. The grids underwent staining in a concentrated alcoholic solution of 2% uranyl acetate for 8 min in a dark setting, followed by three rinses in 70% alcohol, three rinses in ultrapure water, and an 8-min staining with a 2.6% lead citrate solution in an environment free of carbon dioxide. Subsequently, they were rinsed three times with ultrapure water and gently dried using filter paper.

The sections were placed in a grid box and allowed to air dry at room temperature overnight. An HT7800/HT7700 transmission electron microscope (Hitachi High-Technologies Corporation, Tokyo, Japan) was utilized for observations, and Adobe Photoshop 2025 (v26.3.0.156) (Adobe Inc., San Jose, CA, USA) was employed to adjust brightness and contrast in the micrographs, improving the visibility of the key areas of interest [28].

### 2.3. Data Collection

#### 2.3.1. Oil Droplet Characterization

The colors of oil droplets vary based on their focus due to their refractive characteristics [32]. To ensure precise classification, real-time imaging was conducted using a light microscope paired with Motic Images Plus 3.1 (x64) software, with a focus adjustment feature. At a magnification of 40×, the retina was segmented into six distinct areas: the central (CC), temporal (T), dorsal (D), nasal (N), ventral (V), and a randomly chosen ventrotemporal (VT) region [12,33]. For each of these areas, three images of oil droplets, each measuring 0.3 × 0.3 mm^2^, were captured and combined to create a single composite image that encompassed an area of 0.9 × 0.9 mm^2^ [34,35].

Following the classification of oil droplets, the manual cell counter feature in ImageJ v1.54p was employed to mitigate any potential sampling errors during quantification, with only brightness adjustments made and no further image alterations applied. In the combined 0.9 × 0.9 mm viewing area, 15 regions of interest (ROIs), each measuring 1 × 10^−3^ mm^2^, were identified using a five-point sampling technique to tally the different types of oil droplets present in each ROI. For every oil droplet type associated with each species, 15 counts were recorded and aggregated to determine the total quantity of oil droplets in the respective retinal area. To assess the size of each oil droplet type across various retinal regions, 15 droplets of each type were randomly chosen from each composite image, and their diameters were measured using the straight-line measurement tool in ImageJ v1.54p [36].

#### 2.3.2. Tissue Section Samples

Retinal assessments and cellular counts were performed on horizontally sectioned ocular tissues, with specimens screened from the collected individuals (*E. roborowskii*, *n* = 8; *P. axillaris*, *n* = 4), in accordance with the experimental protocol reported by Wahle et al. [8]. For each specimen, three to five consecutive sections with preserved morphology were chosen. A section was classified as the central retina when the optic nerve, lens center, and fovea were aligned in the same plane. Whole-eye images were taken at 4× magnification for sections that fulfilled these criteria, while retinal sections featuring the optic nerve or fovea were captured at 10× magnification. The retina was segmented into six distinct regions [12]. Each individual was analyzed using three consecutive sections and five regions from each section. Ultimately, 120 images from *E. roborowskii* (*n* = 8) and 60 images from *P. axillaris* (*n* = 4) were utilized for quantitative evaluation.

In ImageJ v1.54p, the straight-line measurement tool was employed to assess the thickness of the entire retina as well as its distinct layers: retinal pigment epithelium (RPE), visual cell layer (VCL), outer nuclear layer (ONL), outer plexiform layer (OPL), inner nuclear layer (INL), inner plexiform layer (IPL), ganglion cell layer (GCL), and nerve fiber layer (NFL). For each image, three random thickness measurements were recorded and averaged to minimize bias in the results; the data are expressed as mean ± standard deviation (Mean ± SD) [37]. This method produced thickness data for each retinal layer across both species. The same images utilized for measuring thickness were also analyzed to count photoreceptor cells based on their nuclear structure, facilitating a comparison of photoreceptor density between species [1].

#### 2.3.3. Transmission Electron Microscopy Samples

In the longitudinal perspectives, mid-axial sections that cut through the paraboloid, ellipsoid, and outer segment were chosen. The sizes of the inner and outer segments of the photoreceptors were analyzed with ImageJ v1.54p software. For every parameter, the mean of three distinct measurements was calculated and presented as mean ± standard deviation (Mean ± SD) [28].

### 2.4. Data Analysis

Each image was standardized and preprocessed with Adobe Photoshop version 26.3.0. Adjustments of contrast and brightness were performed to enhance retinal structural visibility and guarantee reliable subsequent quantitative analysis. Counting and linear measurements of photoreceptors and oil droplets across distinct retinal regions were completed using ImageJ v1.54f [28].

Data analysis and graph plotting were carried out in GraphPad Prism 10.4.1. The Shapiro–Wilk test and Levene’s test were first used to examine data normality and homogeneity of variance, respectively. Since datasets for oil droplet parameters, retinal thickness, and photoreceptor density deviated from normal distribution, nonparametric statistics were adopted. The Mann–Whitney U test was applied for pairwise interspecies comparisons, and the Kruskal–Wallis H test was used for multi-group comparisons of oil droplet data. All statistical tests were two-tailed with α = 0.05 set as the significance cutoff.

## 3. Results

### 3.1. Oil Droplet Characterization and Analysis

#### 3.1.1. Oil Droplet Characterization

**Figure 3:**
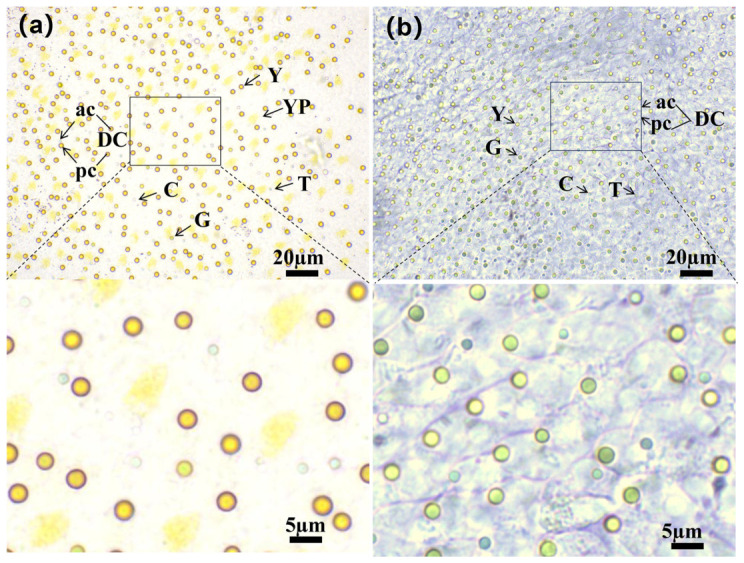
Representative retinal oil droplets of *Eremias roborowskii* (**a**) and *Phrynocephalus axillaris* (**b**). **Note:** Classification of the five oil droplet types: Y = yellow, G = green, C = colorless, T = transparent, pc = principal oil droplet of the double cone, ac = accessory oil droplet of the double cone, YP = diffuse yellow pigment, DC = double cone.

*E. roborowskii* and *P. axillaris* displayed five unique varieties of oil droplets. These were classified according to their appearance into categories: yellow (Y), green (G), transparent (T), colorless (C), and a double cone (DC) which includes a primary (p) and a secondary (a) oil droplet, accompanied by a diffuse yellow pigment (YP) linked to the secondary part of the double cone (Figure 3).

The size of oil droplets differed among various areas of the retina: in the central region, which is rich in photoreceptors, the droplets were smaller compared to those found in the less populated peripheral areas. Y-type droplets exhibited the largest average diameter (*E. roborowskii*: 4.380 ± 0.534 μm; *P. axillaris*: 5.020 ± 0.835 μm), whereas T-type droplets had the smallest average size (*E. roborowskii*: 1.844 ± 0.446 μm; *P. axillaris*: 2.229 ± 0.546 μm). Despite a general trend of decreasing droplet size toward the center of the retina, the average diameter for each type of droplet remained stable across all regions (see Table A1 and Table A2).

#### 3.1.2. Quantitative Analysis of Oil Droplets

All five types of oil droplets were found in every area of the retina, but their distribution and density differed among these regions. In *E. roborowskii*, the central (1904 ± 1449 ind/mm^2^) and temporal (1768 ± 1432 ind/mm^2^) areas exhibited the highest average density for all droplet types, whereas the nasal (1103 ± 777.9 ind/mm^2^) and ventral (1177 ± 949.6 ind/mm^2^) regions had the lowest overall density. For *P. axillaris*, the central area (1385 ± 1452 ind/mm^2^) also recorded the highest average density for each droplet type. Conversely, the Y (2768 ± 926.2 ind/mm^2^) and P (144.4 ± 127.5 ind/mm^2^) types were the least prevalent in the ventrotemporal area, while the nasal region had the fewest C (611.9 ± 239.1 ind/mm^2^), G (1333 ± 679.9 ind/mm^2^), and T (345.0 ± 203.7 ind/mm^2^) droplets (refer to Table A3 and Table A4). The relative proportions of each oil droplet type did not display a consistent trend across the different retinal regions (see Table A5 and Table A6).

**Figure 4:**
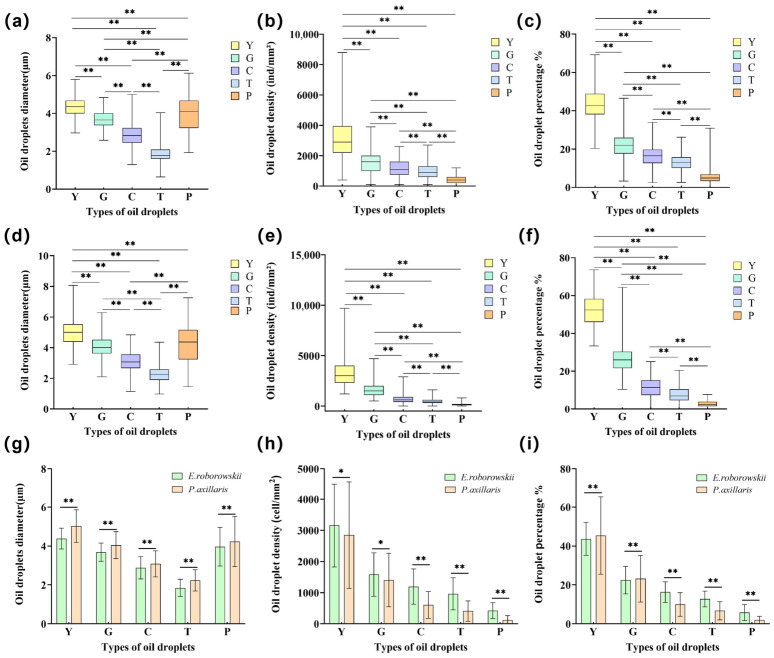
Analysis of oil droplet quantities in *Eremias roborowskii* (**a**–**c**), *Phrynocephalus axillaris* (**d**–**f**), along with comparisons between species (**g**–**i**). **Note**: Statistical evaluations were performed using the Kruskal–Wallis H test, with three biological replicates for each species. Data are presented as mean values along with standard deviation (SD). In panels (**a**,**d**,**g**), the focus is on the diameter of oil droplets; panels (**b**,**e**,**h**) illustrate the density of these droplets, while panels (**c**,**f**,**i**) depict the proportional makeup of the oil droplets. Statistical significance is indicated as follows: * *p* < 0.05, ** *p* < 0.01, and this notation is consistent across all following figures.

The largest droplet sizes were observed in Y-type (*E. roborowskii*: 4.380 ± 0.534 μm; *P. axillaris*: 5.020 ± 0.835 μm) and P-type (*E. roborowskii*: 3.964 ± 0.992 μm; *P. axillaris*: 4.229 ± 1.289 μm) categories, followed by G-type (*E. roborowskii*: 3.683 ± 0.465 μm; *P. axillaris*: 4.040 ± 0.692 μm), C-type (*E. roborowskii*: 2.874 ± 0.568 μm; *P. axillaris*: 3.077 ± 0.671 μm), and T-type (*E. roborowskii*: 1.844 ± 0.446 μm; *P. axillaris*: 2.229 ± 0.546 μm) droplets across both species. The diameter of these droplets is a crucial characteristic for identifying the type of oil droplets within a species. Y-type droplets were found to be the most prevalent in both density and relative abundance, while T-type and P-type droplets were the least frequently observed (Figure 4).

Both species demonstrated a greater density of oil droplets in the dorsal retina (*E. roborowskii*: 1479 ± 1103 ind/mm^2^; *7*: 1246 ± 1338 ind/mm^2^) as well as a higher proportion (*E. roborowskii*: 21.26 ± 15.27; *P. axillaris*: 20.00 ± 19.44) when compared to the ventral retina (*E. roborowskii*: 1177 ± 949.6 ind/mm^2^, 20.0 ± 13.81; *P. axillaris*: 1108 ± 1344 ind/mm^2^, 17.78 ± 19.83).

A comparative analysis indicated that the diameters of all types of oil droplets were notably greater in *P. axillaris* (3.719 ± 1.083 μm) compared to *E. roborowskii* (3.349 ± 1.005 μm). Conversely, *E. roborowskii* exhibited a higher mean density of all oil droplet types (1465 ± 1083 ind/mm^2^) than *P. axillaris* (1081 ± 1101 ind/mm^2^). In terms of oil droplet composition, *P. axillaris* displayed a greater relative abundance of Y-type (45.34 ± 19.87) and G-type (23.15 ± 11.96) droplets compared to *E. roborowskii*. On the other hand, *E. roborowskii* had a higher relative abundance of C-type (16.29 ± 5.304), T-type (12.77 ± 4.010), and P-type (5.791 ± 4.060) droplets than *P. axillaris* (Figure 4i).

### 3.2. Ocular and Retinal Structure

**Figure 5:**
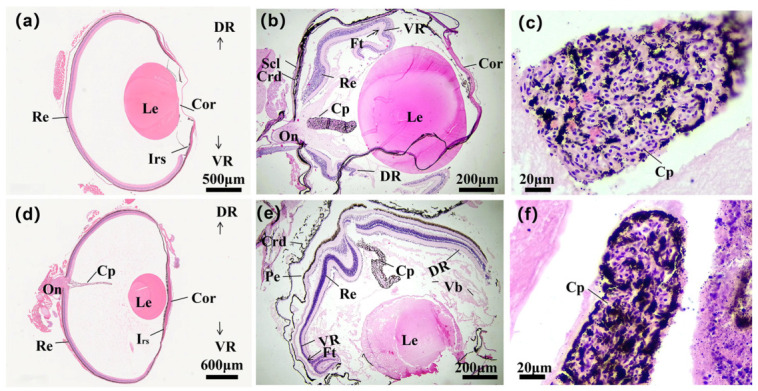
Cross-sectional views of the eyes and conus papillaris. (**a**–**c**) Eye anatomy of Eremias roborowskii: (**a**) Longitudinal slice of the entire eyeball, illustrating the fundamental anatomical features with the dorsal retina (DR) and ventral retina (VR) orientations marked; (**b**) Enlarged depiction of the eye wall and its internal components; (**c**) High-resolution image of the cone papilla (Cp). (**d**–**f**) Eye anatomy of *Phrynocephalus axillaris*: (**d**) Longitudinal slice of the complete eyeball; (**e**) Enlarged view of the eye wall and cone papilla; (**f**) High-resolution image of the cone papilla (Cp). **Abbreviations:** Cor—cornea; Irs—iris; Scl—sclera; Crd—choroid; On—optic nerve; Cp—conus papillaris; Pe—pigment epithelium; Re—retina; Vb—vitreous body; Le—lens; Ft—temporal fovea; DR—dorsal retina; VR—ventral retina. Scale bars: (**a**) = 500 μm, ×2.5; (**d**) = 600 μm, ×2.2; (**b**,**e**) = 200 μm, ×4; (**c**,**f**) = 20 μm, ×40.

The eye structure of *E. roborowskii* and *P. axillaris* includes components such as the cornea, iris, lens, retina, optic nerve, sclera, and choroid. The retina, which has a cup-like shape, covers the inner side of the choroid and partially surrounds the lens. The conus papillaris passes through the optic cup, extends beyond the choroid, and links to the central nervous system (Figure 5b,d,e).

This elongated formation extends toward the lens and is rich in blood vessels, featuring endothelial microplicae and a high concentration of melanocytes packed with melanosomes. Situated in the eye’s midsagittal plane within the vitreous body, this well-vascularized formation provides essential nutrients to the non-vascular retina. The central area of the retina, near the conus papillaris, exhibited the highest thickness and density of photoreceptors. A temporal fovea was observed in the lateral part of the retina in both species.

**Figure 6:**
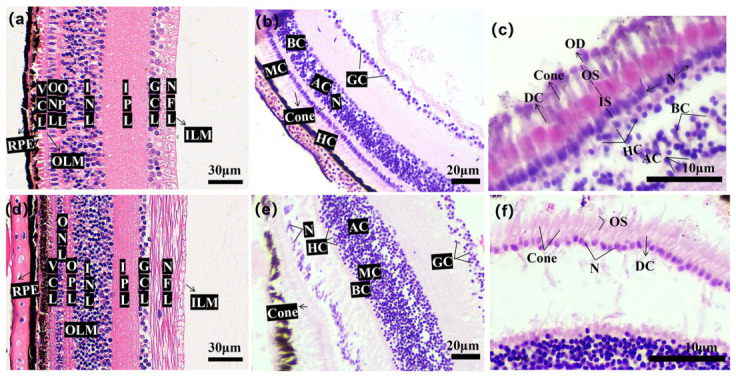
Structure of the retina and photoreceptor cells. (**a**–**c**) Retina of *Eremias roborowskii*: (**a**) Cross-section of the full retinal thickness displaying distinct layers; (**b**) Close-up of the retinal structure and key cell types; (**c**) Magnified view of the photoreceptor layer highlighting the unique shapes of single cones (C) and double cones (DC). (**d**–**f**) Retina of *Phrynocephalus axillaris*: (**d**) Depicting the complete retinal thickness and its layered organization; (**e**) showing the retinal structure and primary cell types; (**f**) High-magnification image of the photoreceptor layer, revealing the distinctive forms of single cones (C) and double cones (DC). **Abbreviations:** The retina consists of ten distinct layers arranged from the outermost to the innermost: RPE—Retinal pigment epithelium; VCL—visual cell layer; OLM—outer limiting membrane; ONL—outer nuclear layer; OPL—outer plexiform layer; INL—inner nuclear layer; IPL—inner plexiform layer; GCL—ganglion cell layer; NFL—nerve fiber layer; ILM—inner limiting membrane. Identified cell types: HC—Horizontal cells; BC—Bipolar cells; AC—Amacrine cells; MC—Müller cells; GC—Ganglion cells. Scale bars and magnifications: (**a**,**d**) = 30 μm, ×40; (**b**,**e**) = 20 μm, ×40; (**c**,**f**) = 10 μm.

*E. roborowskii* and *P. axillaris* both displayed a total of ten unique retinal layers, which can be identified through varying nuclear staining techniques. These layers, arranged from the exterior to the interior, include the RPE, VCL, OLM, ONL, OPL, INL, IPL, GCL, NFL, and ILM (see Figure 6a,d). The outermost layer, the RPE, is made up of a single layer of pigment epithelial cells that feature microvilli extending into the photoreceptor layer, interlocking with the outer segments of the photoreceptors (refer to Figure 6b,e). In *E. roborowskii*, the NFL showed a sparse distribution of nerve fibers, while *P. axillaris* exhibited well-organized bundles of parallel fibers.

Measurements of retinal layer thickness showed no notable differences between species in overall retinal thickness. The IPL was the most substantial layer in both species, with the INL following. *E. roborowskii* exhibited a significantly greater IPL thickness (56.01 ± 14.76 μm) compared to *P. axillaris* (50.76 ± 15.25 μm), which was associated with a higher density of synaptic connections. No significant differences were found in the thickness of the INL and GCL; the GCL consisted of 2–3 layers of cells in both species, while the cells in the INL of *P. axillaris* were arranged more densely (Table 1).

Retinal neurons in both species can be categorized into five main types: photoreceptors, bipolar cells (BC), horizontal cells (HC), amacrine cells (AC), Müller cells (MC), and ganglion cells (GC). These neuron types form three separate nuclear layers: the outer nuclear layer (ONL), These various neuron types create three distinct nuclear layers: the ONL, which contains photoreceptor cells; the INL, whose cells were preliminarily identified as the cell bodies of BC, AC and MC based on their nuclear size, staining intensity, and distribution position, and spatial arrangement; and the ganglion cell layer (GCL), which contains GC. Furthermore, the OPL is situated between the ONL and INL, while the IPL is located between the INL and GCL (Figure 6b,e).

In the layer of photoreceptor cells within the retina, cones are present, with their nuclei mainly found in the ONL. HC, located adjacent to the OPL, display lighter staining and have relatively smaller nuclei. AC, positioned near the IPL, are characterized by a darker stain and more densely packed cell bodies. BC are centrally located in the INL, typically organized in clusters of three to five cell bodies that link neighboring cells; MC, a type of glial cell, extend throughout the INL, with their cell bodies interconnected.

In *E. roborowskii*, the photoreceptors featured a significantly expanded central area, while *P. axillaris* exhibited a unique conical shape. Most photoreceptors in both species contained oil droplets located at the base of the outer segment, which were characterized by a light staining and a semi-transparent appearance (Figure 6c,f).

**Figure 7:**
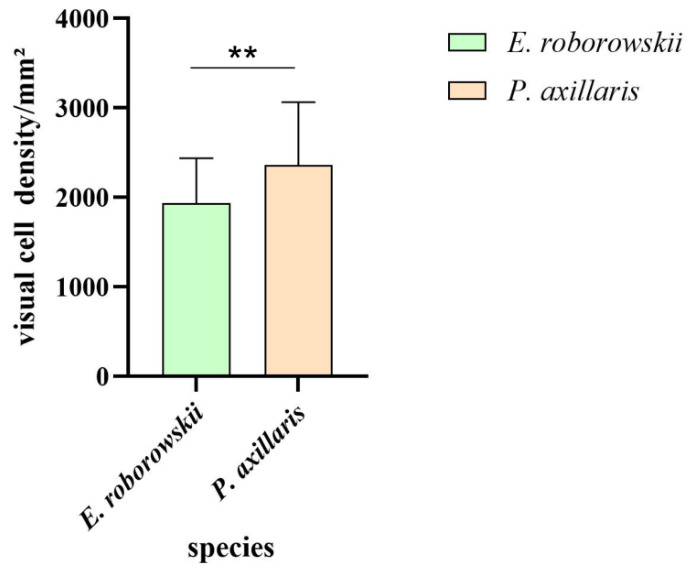
Comparison of photoreceptor cell density between the two lizard species. **Note:** Statistical evaluations utilized the Shapiro–Wilk test to determine normal distribution, subsequently applying the Mann–Whitney U test for comparisons between *E. roborowskii* (*n* = 8) and *P. axillaris* (*n* = 4). Results are shown as mean ± standard deviation (SD). Statistical significance is indicated as follows: ** *p* < 0.01.

The examination of photoreceptor cell density (see Figure 7) revealed that *P. axillaris* exhibited a notably greater density of photoreceptors (2364 ± 701.3 cells/mm^2^) compared to *E. roborowskii*, which had a density of 1936 ± 501.9 inn/mm^2^ (*p* < 0.01).

### 3.3. Photoreceptor Arrangement

**Figure 8:**
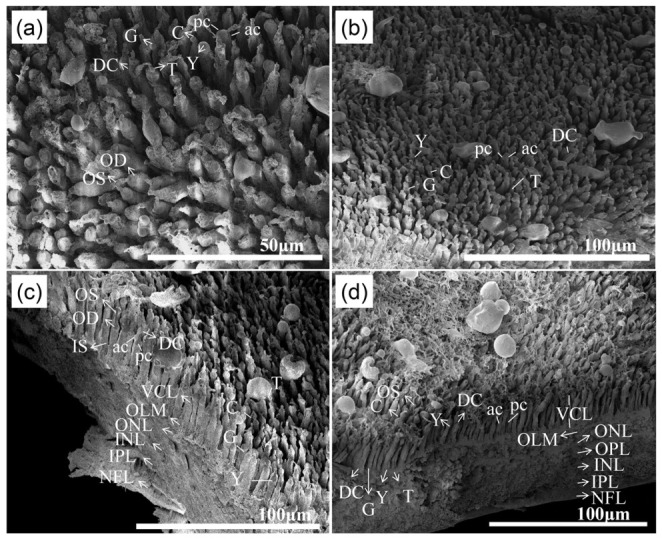
SEM imaging depicting the organization of photoreceptors. (**a**,**c**) *Eremias roborowskii*: (**a**) Frontal perspective of the photoreceptor arrangement displaying the layout of cones; (**c**) Cross-sectional view of the retina depicting the organization of photoreceptors along with the complete layered structure from the outer segments to the nerve fiber layer. (**b**,**d**) *Phrynocephalus axillaris*: (**b**) Frontal perspective of the photoreceptor arrangement; (**d**) Cross-sectional view of the retina. **Abbreviations:** VCL—visual cell layer; OLM—outer limiting membrane; ONL—outer nuclear layer; OPL—outer plexiform layer; INL—inner nuclear layer; IPL—inner plexiform layer; NFL—nerve fiber layer; DC—double cone; OS—outer segment; IS—inner segment; OD—oil droplet; Y—yellow; G—green; C—colorless; T—transparent; p—principal cone of double cone; a—accessory cone of double cone. Scale bars and magnifications: (**a**) = 50 μm, ×1000; (**b**–**d**) = 100 μm, ×500.

SEM revealed that the retinas primarily composed of cone photoreceptors were examined in *E. roborowskii* (Figure 8a,c) and *P. axillaris* (Figure 8b,d). The arrangement of the five cone types was found to be mixed rather than following a uniform mosaic pattern. In line with the findings regarding oil droplets, Y-type cones were the most prevalent throughout the retina, whereas T-type cones were the least common.

In *E. roborowskii* (Figure 8c), the Y-type cones were densely arranged and distinctly structured, featuring clear separations between the inner segments of adjacent cells. Their outer segments were short and rounded, tapering slightly at the tips. Conversely, in *P. axillaris* (Figure 8d), the Y-type cones were arranged more loosely, exhibiting narrower inner segments, while their outer segments retained a conical shape. In both species of daytime lizards, solitary cones (C-type and T-type) were primarily located in the gaps between the Y-type cones, and they were smaller in size compared to the Y-type cones.

### 3.4. Photoreceptor Ultrastructure

**Figure 9:**
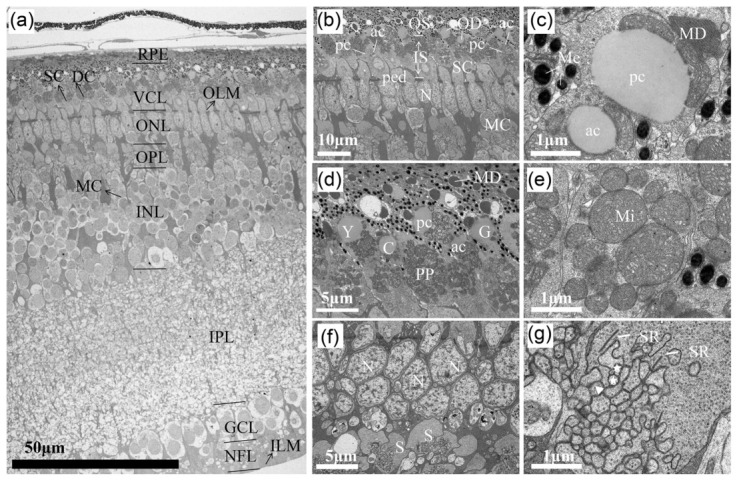
Ultrastructural analysis of the entire retina and photoreceptors in the diurnal *Eremias roborowskii*. (**a**) Longitudinal section of the retina observed via transmission electron microscopy (TEM). (**b**) Morphological characteristics of photoreceptors, detailing their types and composition. (**c**) Structures of oil droplets and membranous disks located in the outer segment of cones. (**d**) Outer and inner segments of cones. (**e**) Enlarged view of mitochondria within the inner segment. (**f**) Nuclei of photoreceptors and synaptic connections in the inner nuclear layer. (**g**) Enlarged depiction of synapses found in the inner plexiform layer. **Abbreviations:** RPE—retinal pigment epithelium; VCL—visual cell layer; OLM—outer limiting membrane; ONL—outer nuclear layer; OPL—outer plexiform layer; INL—inner nuclear layer; IPL—inner plexiform layer; GCL—ganglion cell layer; NFL—nerve fiber layer; ILM—inner limiting membrane; SC—single cone; DC—double cone; OS—outer segment; IS—inner segment; PC—principal cone of the double cone; AC—accessory cone of the double cone; MD—single lamellar membranous disc; PP—paraboloid; ped—photoreceptor pedicle; N—photoreceptor nucleus; Me—melanosome; Mi—mitochondria; G—Golgi apparatus; S—synaptic terminal; SR—cone synaptic ribbon; MC—Müller cells; OD—oil droplet. In panel (**f**), the thick white arrow indicates the synaptic connection between the ONL and OPL. In panel (**g**), the star (☆) marks vesicles in horizontal cell dendrites, and the triangle (△) marks vesicles in bipolar cell dendrites. The same labels apply to subsequent figures. Scale bars and magnifications: (**a**) = 50 μm, ×3000; (**b**) = 10 μm, ×1000; (**c**,**e**,**g**) = 1 μm, ×15,000; (**d**,**f**) = 5 μm, ×2500.

**Figure 10:**
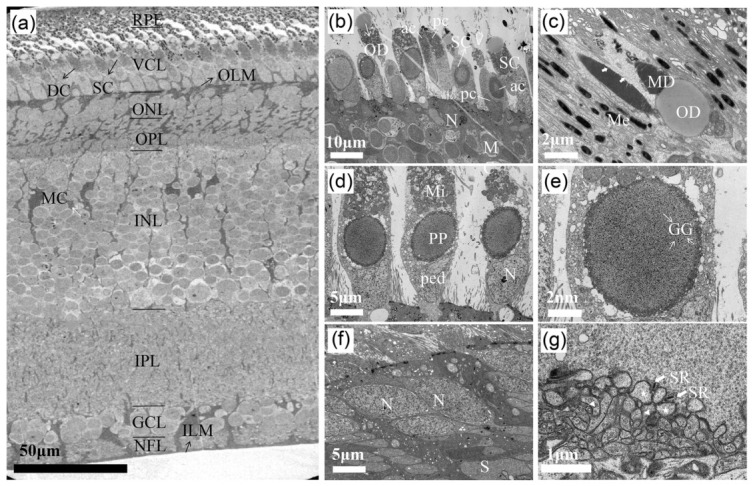
Ultrastructural analysis of the entire retina and photoreceptors in the diurnal *Phrynocephalus axillaris*. (**a**) Longitudinal section of the retina observed via transmission electron microscopy (TEM). (**b**) Morphological characteristics of photoreceptors, detailing their types and composition. (**c**) Structures of oil droplets and membranous disks located in the outer segment of cones. (**d**) Inner segment of cones. (**e**) Enlarged depiction of the paraboloid within the inner segment, rich in glycogen granules. (**f**) Nuclei of photoreceptors situated in the inner nuclear layer. (**g**) Enlarged view of synaptic connections in the inner plexiform layer. **Abbreviations:** GG—glycogen granules. In panel (**g**), the star (☆) marks vesicles in horizontal cell dendrites, and the triangle (△) marks vesicles in bipolar cell dendrites. Scale bars and magnifications: (**a**) = 50 μm, ×300; (**b**) = 10 μm, ×1000; (**c**) = 2 μm, ×5000; (**d**) = 5 μm, ×2000; (**e**) = 2 μm, ×5000; (**f**) = 5 μm, ×2500; (**g**) = 1 μm, ×15,000.

The VCL in both *E. roborowskii* and *P. axillaris* features both single and double cones, with oil droplets positioned at the interface of the inner and outer segments (see Figure 9 and Figure 10b). These oil droplets are smooth and lack any visible internal structure. Beyond the oil droplets, the outer segment consists of interconnected single lamellar discs that are partially surrounded by the outer mitochondrial membrane of the ellipsoid (refer to Figure 9 and Figure 10c). In *E. roborowskii*, the ellipsoid of each cone cell is densely populated with mitochondria that increase in size from the edges toward the center, nearly occupying the entire inner segment. As a result, glycogen granules in the paraboloid are limited, being more prevalent in the principal cone of the double cones (see Figure 9d,e). In *P. axillaris*, the arrangement of single and double cones forms a distinctive mosaic pattern. Within the double cones, both the principal and accessory components are of similar size; each cone has oil droplets of varying sizes on its apical surface, and the outer segments, along with stacked lamellar discs, are positioned directly above the oil droplets. Each photoreceptor contains mitochondria and an elliptical paraboloid filled with dense glycogen granules, with a more irregular distribution of mitochondria compared to *E. roborowskii* (Figure 10b–e).

In *E. roborowskii*, the ONL features 2 to 3 layers of photoreceptor nuclei of varying sizes, organized in a staggered formation just above the OLM. In contrast, *P. axillaris* has an ONL comprising 1 to 2 layers of photoreceptor nuclei, also arranged in a staggered manner next to the OLM, with some nuclei extending through this membrane. Beneath each row of nuclei, synaptic terminals are located, creating clusters that include synaptic ribbons and forming numerous connections with postsynaptic neurons (Figure 9 and Figure 10e,f). The photoreceptor nuclei establish synaptic links with neurons in the INL through synaptic ribbons at their terminals (Figure 10f,g).

In *E. roborowskii*, the INL consisted of 7 to 8 layers of cells, whereas *P. axillaris* exhibited 10 to 11 layers, suggesting that the latter has a greater density of neuronal connections (Figure 9 and Figure 10a). The MC spanned the full extent of both the INL and ONL (Figure 10a). Supporting findings from histological analyses, TEM imaging showed a greater number of neuronal connections in *E. roborowskii* compared to *P. axillaris*.

In *P. axillaris*, the INL comprised 10 to 11 layers of cells, suggesting a greater density of neuronal connections compared to *E. roborowskii*. The MC spanned both the INL and the ONL (see Figure 10a). The arrangement of single and double cones in *P. axillaris* resembled a mosaic. In the case of double cones, both the principal and accessory cones were of comparable size, each containing an oil droplet of varying dimensions, with the outer segment of the cone and circular membranous discs positioned directly above the oil droplet. Each cone was equipped with mitochondria and an elliptical paraboloid filled with dense glycogen granules (refer to Figure 10b–e). The ONL featured 1 to 2 layers of photoreceptor nuclei that established synaptic connections with neurons in the INL through synaptic ribbons at their terminals (illustrated in Figure 10f,g).

## 4. Discussion

### 4.1. Inter-Specific Differences and Functional Adaptation of Oil Droplets

In the photoreceptor cells of various reptiles, a distinctive structure known as the oil droplet is present. These droplets are found in turtles and most lizards, but are absent in crocodiles, geckos, and snakes [38]. Oil droplets are crucial for improving color discrimination by limiting the spectral sensitivity of visual pigments in the outer segments of cones [39]. Their elevated refractive index aids in capturing photons by directing incoming light onto the outer segment [40]. Victory et al. [35,41] examined five adult yellow-legged gulls (*Larus michahellis*) and confirmed that their retinas possess a double fovea and five types of cone oil droplets. The oil droplets show a central high-density horizontal band and a dorsoventral gradient, specializations that are adapted to ecological needs such as foraging and navigation in open seas.

In fresh retinal flat-mounts from the diurnal lizards *E. roborowskii* and *P. axillaris*, we discovered five different types of colored oil droplets (yellow, green, colorless, transparent, and double-cone droplets) along with diffuse yellow pigment (YP), aligning with findings in other lizard species [18]. Prior research suggests that the various types of single-cone oil droplets (Y-type, G-type, T-type, and C-type) and P-type double-cone oil droplets correspond to specific photoreceptors (LWS, MWS, UVS, SWS1, and LWS double-cone) [12,35,42,43,44]. Based on earlier studies, we deduce that the oil droplets in *E. roborowskii* and *P. axillaris* are linked to cone visual pigments with different spectral sensitivities, indicating that these lizard species likely have an advanced color vision system vital for their foraging activities. Furthermore, the transparent T-type oil droplets in squamate cones permit ultraviolet (UV) wavelengths to pass, suggesting the existence of UV-sensitive photoreceptors in the retinas of both species.

In addition to their hues, oil droplets can be distinguished by their dimensions. Mariani & Leure-Dupree [45] were pioneers in correlating the coloration of oil droplets with their size in the retinas of pigeons, demonstrating that droplets that capture longer wavelengths tend to be larger than those that capture shorter wavelengths. Our comprehensive analysis of oil droplet sizes, both within and across species, revealed that Y-type and P-type droplets were the largest in both *E. roborowskii* and *P. axillaris*, followed by G-type and T-type droplets, while C-type droplets were the smallest. The larger droplets were located in the peripheral retina, which has a lower density of photoreceptors, whereas the smaller droplets were found in the central retina, where photoreceptor density is higher. The combination of larger oil droplets and shorter outer segments improves light absorption [35,46], potentially accounting for the increased droplet size in the peripheral retina of both species. The double cone droplets have a low carotenoid concentration and absorb light within the 420–480 nm spectrum [47,48], which is shorter than that of other oil droplet types. In both species studied, the accessory cones of double cones contained oil droplets that appeared pale green under light microscopy, aligning with the spectral characteristics of MWS cones.

Differences in the density and relative amounts of cones significantly influence how animals perceive colors. In our study of six retinal areas in *E. roborowskii*, we observed notable differences in the total number of oil droplets and their types. The central and temporal regions exhibited the highest concentrations of each droplet type, whereas the nasal region had fewer, and the ventral region showed the least amount of double cones. Interestingly, *E. roborowskii* displayed a greater density and proportion of oil droplets in the dorsal retina compared to the ventral area, contrasting with the findings for *P. axillaris*. This increase in oil droplet density from dorsal to anterior regions corresponds with the existence of a visual streak (VS) in the back of the eye [35]. According to the Retinal Topography Theory [49], this characteristic is common among species that thrive in open habitats, aligning with the ecological context of *P. axillaris* in our research. Regions of the retina with a high concentration of photoreceptors are linked to improved spatial resolution, which aids in functions like motion detection and spatial awareness [35,41,50]. The abundance of Y-type oil droplets, which serve as filters for long wavelengths, likely enhances the ability to detect prey in both species. *E. roborowskii* is mainly found in shrubland environments, and based on earlier studies, we hypothesize that the increased oil droplet density in its dorsal retina may enhance the contrast and clarity of objects observed on the ground [51].

The distribution of different types of oil droplets showed notable variation among the six regions of the retina, aligning with the observed density patterns. Each retinal area within a species exhibited differences in the total count of oil droplet types; however, the ratios of these droplet types remained stable across all regions in *Anolis* lizards [12]. This observation highlights the necessity of a balanced cone type ratio for effective visual processing [12]. Our research indicated that *E. roborowskii* had significantly greater relative amounts of C-type, T-type, and P-type oil droplets compared to *P. axillaris*, while the latter species exhibited higher proportions of Y-type and G-type droplets. This pronounced difference in oil droplet composition between the two species likely indicates adaptations to varying light environments and visual requirements. The differences in oil droplet ratios among species are influenced by the spectral characteristics of their respective light habitats [12]. Despite both species being active during the day, their unique microhabitats and foraging behaviors imply that specific visual tasks, rather than merely the surrounding light conditions, are key factors influencing the quantity and ratio of oil droplets.

### 4.2. Retinal Structural Traits and Their Ecological Adaptations

The eye structure of the two lizard species adheres to the fundamental design typical of reptiles, comprising elements such as the cornea, iris, lens, and retina (see Figure 6a,b), which aligns with findings from other lizard studies [31]. Research conducted by Yovanovich et al. [52] on the burrowing lizard *Calyptommatus nicterus* revealed that, although its eye is quite small (~600 μm in diameter), its retinal composition closely resembles that of the diurnal lizard *Ameivula ocellifera*. Both species exhibit a complete vertebrate retinal structure, including the retinal pigment epithelium (RPE). The RPE in these lizards is densely filled with melanin granules, which effectively absorb stray light and enhance visual contrast, showcasing a common adaptive feature among lizards inhabiting arid environments with intense light conditions [52]. This suggests that the fundamental arrangement of retinal tissues is remarkably stable, ensuring essential visual capabilities.

Our research discovered a temporal fovea in both *E. roborowskii* and *P. axillaris* (see Figure 5b,e). This observation aligns with the traditional theories proposed by Walls [38] and Röll [31], which indicate that nocturnal animals often forfeit the fovea to enhance light sensitivity, whereas many daytime lizards exhibit a temporal fovea. Canei et al. [1] noted that two burrowing psammophilic skinks (*Scincus scincus* and *Eumeces schneideri*) do not possess a fovea; despite being mainly diurnal, their extended time spent in subterranean sandy environments has resulted in a visual system that favors light sensitivity over sharp visual detail. Consequently, our findings imply that the existence of a temporal fovea in the diurnal lizards *E. roborowskii* and *P. axillaris* is likely an evolutionary adaptation to facilitate enhanced visual acuity while foraging in daylight.

According to Röll [31], the temporal fovea is situated in the front part of the lateral eye, aligning with the center of the lizard’s binocular vision. Upon spotting prey, the lizard positions its head and eyes to focus on the target using both eyes, ensuring that the image of the prey within striking range is sharply focused on the temporal fovea [31]. This feature aligns with the ecological needs of daytime hunting lizards like *E. roborowskii* and *P. axillaris*. In a similar vein, Nagloo et al. [53] noted similar retinal features in two species of Australian crocodiles, which have a central fovea and a visual streak. These adaptations enable crocodiles to spot potential prey on the riverbank with excellent spatial resolution while staying hidden. The placement of these retinal adaptations is closely associated with the ecological roles and foraging behaviors of each species.

In our examination of both species, we noted a prominent conus papillaris (Cp) at the optic nerve head (Figure 5c,f), which is a richly vascularized formation that provides essential nutrients to the non-vascular retina [31]. New et al. [5] detailed the Cp in the skink *Tiliqua rugosa*, highlighting an intricate arrangement of capillaries and larger blood vessels mixed with melanocytes and connective tissue, aligning with our findings in the two lizard species.

Notably, the IPL of *E. roborowskii* was significantly thicker than that of *P. axillaris*, suggesting a more complex neuronal network to support the detection of moving insect prey during active foraging [14,53]. The developmental degree of the NFL exhibits interspecific variations consistent with those of the ONL, with well-defined thick parallel fiber bundles observed in *P. axillaris* relative to *E. roborowskii*. This implies that ganglion cells in *P. axillaris* possess more abundant axons, enabling more efficient transmission of visual signals toward the central nervous system, an adaptive feature matching its behavioral traits of rapid locomotion, predation, and predator avoidance [1].

No notable statistical differences were found in the overall retinal thickness between the two lizard species. Upon analyzing the retinal layers, the INL and the IPL comprised the largest portions, with the INL measuring between 21.75% and 23.90% and the IPL ranging from 23.36% to 28.00%. The IPL serves as the main area for synaptic integration among BC, AC, and GC, and its thickness is closely linked to the number of synapses and the density of neural circuits. A thicker IPL indicates a greater capacity for synaptic integration and a more sophisticated processing of visual information [54]. Interestingly, the IPL in *E. roborowskii* was significantly thicker compared to that in *P. axillaris*, implying a more intricate neuronal network that aids in detecting moving insect prey during active foraging [14,53]. The development of the NFL shows interspecific differences that align with those of the ONL, with *P. axillaris* displaying well-defined, thick parallel fiber bundles in contrast to *E. roborowskii*. This suggests that ganglion cells in *P. axillaris* have a higher number of axons, facilitating more effective transmission of visual signals to the central nervous system, which is an adaptive trait suited to its behaviors of swift movement, hunting, and evading predators [1].

### 4.3. Photoreceptor Arrangement and Ultrastructure

Our study revealed five unique types of cones in the retinas of the daytime lizards *E. roborowskii* and *P. axillaris*, categorized by the color of their oil droplets: yellow (Y), green (G), colorless (C), and transparent (T) single cones, along with double cones (P) (see Figure 7). These cones did not form a regular geometric pattern. A similar observation was made by Barbour et al. [15] in the daytime lizard *Ctenophorus ornatus*, which exhibited a ratio of double cones to single cones of approximately 1:4, lacking a highly organized mosaic and featuring double cones mixed among single cones. This configuration might enhance the uniformity of light sampling by photoreceptors, rather than facilitating specialized spatial frequency channels [15]. In *C. ornatus*, Barbour et al. [15] noted that the principal and accessory cones of the double cones are intertwined, with the accessory cone possessing a shorter inner segment, resulting in the outer segments of both components rarely appearing in the same longitudinal section. This could account for the fragmented look of double cone outer segments observed in our TEM studies. The exact function of double cones is still a topic of discussion. Barbour et al. [15] proposed that they might play a role in motion detection in lizards, akin to the function of double cones in birds, which are recognized for their role in motion perception [32]. As typical diurnal desert lizards, *E. roborowskii* and *P. axillaris* are active during the day when light is plentiful and depend solely on photopic vision. Their short, conical outer segments reduce self-screening of visual pigments, enhance the signal-to-noise ratio, and allow for more effective light focusing onto the outer segment through the ellipsoid [13]. This structural characteristic aligns with the adaptation of these two diurnal lizard species to fluctuating ambient light conditions.

The ellipsoid located in the inner segment of photoreceptor cells consists of densely arranged mitochondria and is essential for energy generation and light detection [15]. In our study of *E. roborowskii* and *P. axillaris*, we noted a variation in mitochondrial dimensions within the ellipsoid, with the central region containing larger mitochondria and the outer areas housing smaller ones. This arrangement aligns with findings from El-Bakary et al. [14] regarding the African five-lined skink and could create a gradient in refractive index that improves light delivery to the visual pigment layer in the outer segment.

Elevated mitochondrial concentrations in the ellipsoid of *E. roborowskii* could enhance light absorption and processing in bushy environments. Conversely, the advanced paraboloid structure of *P. axillaris* allows for adjustments in the angle and strength of incoming light in exposed areas with high sunlight exposure. The structural differences in ellipsoids and paraboloids between these two lizard species may indicate visual adaptations that align with their unique microhabitats.

### 4.4. Limitations and Future Perspectives

This research conducted a thorough comparison of the retinal structure and ultrastructure of two lizard species that coexist in the same habitat, yielding essential baseline information for studies on visual adaptation in reptiles inhabiting arid environments. However, the study faced several challenges due to the limitations of field sampling wild lizards. Owing to the limited availability of wild populations of the target species, the present study was conducted with a relatively modest sample size, which might to a certain degree affect the statistical power of the analyses [41,55]. Additionally, the nature of field collection hindered the ability to control for confounding factors such as sex, age, body weight, and the season of sampling among individuals [37]. The lack of in situ measurements of ambient light in their microhabitats means that the proposed relationship between retinal structure and local light conditions is based solely on morphological data rather than confirmed by environmental field data [56,57].

Furthermore, our analysis of phenotypic variation is based only on light and electron microscopy; without additional assessments of opsin expression, spectral sensitivity, and visual performance, the adaptive importance of retinal differences cannot be fully understood at the molecular and functional levels. Given the significant genetic divergence between the two species studied, this research primarily emphasizes retinal adaptations driven by microhabitat conditions rather than disentangling phylogenetic influences. The concepts of phylogenetic history and habitat adaptation are separate areas of study that do not contradict each other [58], which is why phylogenetic effects were not the main focus of this research. Despite the limitations associated with field sampling, this study contributes valuable baseline data on the retinal microstructure and ultrastructure of the two desert lizard species, serving as a useful reference for future related research. Subsequent studies could further explore the evolutionary processes behind retinal light adaptation by broadening sampling efforts, standardizing individual biological factors, performing in situ light assessments, integrating molecular and functional analyses, and incorporating phylogenetic studies [3].

## 5. Conclusions

In summary, this study systematically compared interspecific differences in retinal morphology, oil droplet traits, photoreceptor arrangement and ultrastructure between two sympatric lizards (*E. roborowskii* and *P. axillaris*) inhabiting contrasting microhabitats in the Turpan Basin. Both species have cone-dominated retinas featuring a temporal fovea, vascularized conus papillaris and five types of pigmented oil droplets, typical adaptive structures for diurnal vision in desert lizards. Distinct interspecific variations in oil droplet size, density, and proportion, as well as divergent ultrastructural morphology of photoreceptor ellipsoids and paraboloids, were observed between the two species. Based on their known microhabitat differences, these retinal disparities likely reflect adaptive differentiation corresponding to divergent light environments. The open-terrain *P. axillaris* develops larger oil droplets and glycogen-rich paraboloids, which are morphologically conducive to coping with high-intensity solar radiation. In contrast, shrub-dwelling *E. roborowskii* exhibits denser oil droplets and mitochondria-abundant ellipsoids, structural traits that may facilitate the capture and utilization of diffuse ambient light. Such phenotypic retinal differences are presumed to be adaptive outcomes shaped by microhabitat divergence and distinct foraging strategies. This study provides fundamental morphological data for further investigations into visual adaptation in desert reptiles.

## Figures and Tables

**Table 1:** Thickness of individual retinal layers in *Eremias roborowskii* and *Phrynocephalus axillaris*.

Retinal Layer Thickness	*Eremias roborowskii* (μm)	*Phrynocephalus axillaris* (μm)	*p*-Value
RPE	15.97 ± 4.02	18.16 ± 6.46	0.16
VCL	24.60 ± 9.45	22.49 ± 6.09	0.41
ONL	10.31 ± 3.27	10.09 ± 3.63	0.34
OPL	13.21 ± 8.09	14.58 ± 6.28	0.15
INL	44.53 ± 14.24	46.55 ± 16.90	0.74
IPL	56.01 ± 14.76	50.76 ± 15.25	0.01
GCL	12.14 ± 6.10	11.92 ± 3.22	0.44
NFL	24.89 ± 7.102	24.45 ± 5.20	0.69
Total retinal thickness	202.5 ± 33.99	199.0 ± 50.10	0.23

**Abbreviations:** RPE—retinal pigment epithelium; VCL—visual cell layer; ONL—outer nuclear layer; OPL—outer plexiform layer; INL—inner nuclear layer; IPL—inner plexiform layer; GCL—ganglion cell layer; NFL—nerve fiber layer.

## Data Availability

The dataset is available upon request from the authors.

## Abbreviations

The following abbreviations are used in this manuscript:

Cor	cornea
Irs	iris
Scl	sclera
Crd	choroid
On	optic nerve
Cp	conus papillari
Pe	pigment epithelium
Re	retina
Vb	vitreous body
Le	lens
Ft	temporal fovea
DR	dorsal retina
VR	ventral retina
RPE	Retinal pigment epithelium
VCL	visual cell layer
OLM	outer limiting membrane
ONL	outer nuclear layer
OPL	outer plexiform layer
INL	inner nuclear layer
IPL	inner plexiform layer
GCL	ganglion cell layer
NFL	nerve fiber layer
ILM	inner limiting membrane
Cone	Cone cells
CBC	Cone bipolar cells
HC	Horizontal cells
BC	Bipolar cells
DBC	Displaced bipolar cells
AC	Amacrine cells
MC	Müller cells
GC	Ganglion cells
SC	single cone
DC	double cone
OS	outer segment
IS	inner segment
OD	oil droplet
Y	Yellow oil droplet
G	Green oil droplet
C	Colorless oil droplet
T	Transparent oil droplet
pc	principal cone of double cone
ac	accessory cone of double cone
MD	single lamellar membranous disc
PMD	parallel membranous disc
EMD	elongated membranous disc
E	ellipsoid
PP	paraboloid
ped	photoreceptor pedicle
N	photoreceptor nucleus
Me	melanosome
Mi	mitochondria
G	Golgi apparatus
S	synaptic terminal
SR	cone synaptic ribbon
GG	glycogen granules
CC	central region
T	temporal region
D	dorsal region
N	nasal region
V	ventral region
VT	ventrotemporal region

## Appendix A

**Table A1:** Difference analysis of oil droplet diameters (µm, Mean ± SD) in the 6 regions of the retina of *Eremias roborowskii*.

Area	Y-Type	G-Type	C-Type	T-Type	P-Type
CC	4.523 ± 0.460 ^a,b^	3.794 ± 0.431 ^a,b^	2.964 ± 0.423 ^a^	1.881 ± 0.357 ^a^	3.808 ± 1.117 ^a^
T	4.285 ± 0.456 ^a,b^	3.535 ± 0.415 ^B,b^	3.026 ± 0.592 ^a^	1.699 ± 0.358 ^a,c^	3.909 ± 1.036 ^a^
D	4.425 ± 0.506 ^a,b^	3.711 ± 0.340 ^a,b^	3.057 ± 0.560 ^a,b^	1.978 ± 0.366 ^a,b^	4.125 ± 0.807 ^a^
N	4.119 ± 0.339 ^B^	3.480 ± 0.332 ^B,b^	2.593 ± 2.898 ^a,B,c^	1.580 ± 0.432 ^B,C^	3.791 ± 0.871 ^a^
V	4.580 ± 0.551 ^a,b^	3.951 ± 0.435 ^a,b^	2.898 ± 0.613 ^a^	1.982 ± 0.578 ^a^	4.318 ± 1.042 ^a^
VT	4.347 ± 0.712 ^a,b^	3.629 ± 0.628 ^b^	2.708 ± 0.598 ^a^	1.942 ± 0.403 ^a^	3.832 ± 0.985 ^a^

Notes: Significant differences between groups are indicated by letter markings. Within the same column comparison, values sharing the same lowercase letter are not significantly different (*p* > 0.05), while different lowercase letters indicate a significant difference (*p* < 0.05). Different uppercase letters correspond to a highly significant difference (*p* < 0.01). The letter markings are assigned based on the descending order of mean values. This annotation method applies to the following tables as well.

**Table A2:** Difference analysis of oil droplet diameters (µm, Mean ± SD) in the 6 regions of the retina of *Phrynocephalus axillaris*.

Area	Y-Type	G-Type	C-Type	T-Type	P-Type
CC	4.641 ± 0.894 ^B^	3.748 ± 0.783 ^B^	2.861 ± 0.834 ^a,b^	2.010 ± 0.404 ^B^	4.207 ± 1.169 ^a^
T	4.905 ± 0.541 ^a^	3.996 ± 0.588 ^a^	2.944 ± 0.807 ^a^	2.150 ± 0.355 ^B^	4.299 ± 1.051 ^a^
D	4.769 ± 0.617 ^b^	3.943 ± 0.370 ^a^	2.899 ± 0.466 ^b^	1.985 ± 0.373 ^B,c^	3.916 ± 1.389 ^a^
N	5.123 ± 0.728 ^a^	4.038 ± 0.666 ^a^	3.171 ± 0.537 ^a^	2.290 ± 0.691 ^a,c^	4.144 ± 1.432 ^a^
V	5.376 ± 0.854 ^a^	4.215 ± 0.877 ^a^	3.231 ± 0.635 ^a^	2.591 ± 0.582 ^a^	4.544 ± 1.208 ^a^
VT	5.306 ± 1.039 ^a^	4.302 ± 0.647 ^a^	3.349 ± 0.538 ^a^	2.347 ± 0.558 ^a^	4.264 ± 1.423 ^a^

Notes: See Table A1 for explanations of lowercase and uppercase letter markings.

**Table A3:** Difference analysis of oil droplet density (ind/mm^2^, Mean ± SD) in the 6 regions of the retina of *Eremias roborowskii*.

Area	Y-Type	G-Type	C-Type	T-Type	P-Type
CC	4144 ± 1255 ^a^	2118 ± 662.4 ^a^	1509 ± 628.8 ^a^	1251 ± 629.1 ^a^	497.8 ± 278.4 ^a^
T	4047 ± 1375 ^a^	1749 ± 713.4 ^a^	1411 ± 513.6 ^a,b^	1167 ± 428.5 ^a,b^	468.9 ± 226.5 ^a,b^
D	3169 ± 975.6 ^a,b^	1649 ± 575.9 ^a^	1056 ± 507.9 ^b^	1009 ± 432.1 ^a,b^	448.9 ± 254.6 ^a,b^
N	2093 ± 966.8 ^B^	1098 ± 601.3 ^B^	904.4 ± 474.8 ^B^	637.8 ± 374.9 ^B^	442.2 ± 604.3 ^a,b^
V	2453 ± 1086 ^B^	1211 ± 609.9 ^B,b^	1056 ± 464.4 ^B^	726.7 ± 362.7 ^B^	282.2 ± 220.8 ^B^
VT	2544 ± 1329 ^B^	1382 ± 756.3 ^a,B^	1027 ± 701.4 ^b^	817.8 ± 618.8 ^B^	315.6 ± 229.6 ^b,c^

Notes: See Table A1 for explanations of lowercase and uppercase letter markings.

**Table A4:** Difference analysis of oil droplet density (ind/mm^2^, Mean ± SD) in the 6 regions of the retina of *Phrynocephalus axillaris*.

Area	Y-Type	G-Type	C-Type	T-Type	P-Type
CC	3540 ± 1453 ^a^	1896 ± 830.7 ^a^	797.3 ± 367.0 ^a^	570.3 ± 297.1 ^a^	191.9 ± 123.3 ^a^
T	3379 ± 1352 ^a^	1674 ± 658.5 ^a^	611.9 ± 239.1 ^a^	504.2 ± 358.1 ^a^	184.8 ± 137.2 ^a^
D	3384 ± 1267 ^a^	1582 ± 556.9 ^a^	635.6 ± 303.1 ^a^	502.2 ± 266.7 ^a^	173.5 ± 79.04 ^a^
N	3338 ± 2113 ^a^	1333 ± 679.9 ^b^	775.0 ± 588.5 ^a^	345.0 ± 203.7 ^b^	246.7 ± 238.6 ^a^
V	3380 ± 1229 ^a^	1658 ± 727.1 ^a^	642.5 ± 413.8 ^a^	402.5 ± 285.1 ^a^	147.5 ± 117.6 ^a^
VT	2768 ± 926.2 ^a^	1484 ± 626.5 ^a^	777.8 ± 391.3 ^a^	555.6 ± 352.0 ^a,b^	144.4 ± 127.5 ^a^

Notes: See Table A1 for explanations of lowercase and uppercase letter markings.

**Table A5:** Difference analysis of oil droplet percentage (%, Mean ± SD) in the 6 regions of the retina of *Eremias roborowskii*.

Area	Y-Type	G-Type	C-Type	T-Type	P-Type
CC	44.04 ± 5.700 ^a^	22.88 ± 5.249 ^a,b^	15.41 ± 3.281 ^a^	12.53 ± 3.809 ^a^	5.148 ± 2.993 ^a^
T	46.10 ± 8.534 ^a^	19.38 ± 4.626 ^B^	16.10 ± 4.623 ^a^	12.98 ± 3.278 ^a^	5.439 ± 2.488 ^a^
D	44.08 ± 10.25 ^a^	27.78 ± 10.182 ^a^	14.09 ± 5.425 ^B,b^	13.55 ± 4.164 ^a^	6.115 ± 2.905 ^a^
N	41.13 ± 7.654 ^a^	20.91 ± 5.602 ^b^	17.70 ± 5.397 ^a^	12.22 ± 4.068 ^a^	8.038 ± 6.994 ^a^
V	42.51 ± 7.848 ^a^	20.86 ± 5.874 ^b^	18.95 ± 5.918 ^a^	12.72 ± 4.094 ^a^	4.958 ± 4.111 ^a^
VT	43.75 ± 9.801 ^a^	22.92 ± 6.380 ^a,b^	15.57 ± 5.589 ^a^	12.54 ± 4.682 ^a^	5.215 ± 2.580 ^a^

Notes: See Table A1 for explanations of lowercase and uppercase letter markings.

**Table A6:** Difference analysis of oil droplet percentage (%, Mean ± SD) in the 6 regions of the retina of *Phrynocephalus axillaris*.

Area	Y-Type	G-Type	C-Type	T-Type	P-Type
CC	50.89 ± 7.161 ^a^	27.23 ± 5.273 ^a^	13.76 ± 3.912 ^a^	9.843 ± 4.022 ^a^	2.784 ± 1.749 ^a^
T	54.03 ± 8.939 ^a^	27.05 ± 6.683 ^a^	10.67 ± 4.640 ^a,b^	8.674 ± 3.678 ^a^	3.099 ± 1.649 ^a^
D	53.64 ± 19.97 ^a^	25.35 ± 5.763 ^a^	10.39 ± 4.578 ^a,b^	8.612 ± 4.713 ^a^	2.911 ± 1.535 ^a^
N	53.0 ± 8.462 ^a^	29.24 ± 16.50 ^a^	13.51 ± 5.591 ^a^	5.963 ± 3.406 ^B^	2.866 ± 1.888 ^a^
V	54.61 ± 9.657 ^a^	26.23 ± 5.856 ^a^	10.39 ± 4.881 ^a,b^	6.485 ± 3.174 ^B^	2.291 ± 1.425 ^a^
VT	48.67 ± 7.122 ^a,b^	25.37 ± 6.529 ^a^	11.62 ± 5.149 ^a^	7.969 ± 4.114 ^a^	2.368 ± 1.785 ^a^

Notes: See Table A1 for explanations of lowercase and uppercase letter markings.

**Table A7:** Basic sample information and experimental allocation of *Eremias roborowskii* and *Phrynocephalus axillaris*.

Species	ID	Sex	Age Class	SVL (mm)	Body Mass (g)	Sampling Locality	Experimental Usage	Number of Individuals	Number of Eyeballs
*Eremias roborowskii*	E-01	M	Adult	65.56	5.81	Toksun County	SEM, TEM	3	3, 3
	E-02	M	Adult	66.04	6.48	Toksun County	SEM, TEM		
	E-03	F	Adult	67.24	7.93	Toksun County	SEM, TEM		
	E-04	F	Adult	68.07	7.64	Toksun County	OD, HE	3	4, 2
	E-05	F	Adult	63.35	6.09	Toksun County	OD, HE		
	E-06	M	Adult	65.84	7.31	Toksun County	OD, HE		
	E-07	F	Adult	57.49	4.89	Toksun County	HE	6	6/12
	E-08	F	Adult	68.91	8.34	Toksun County	HE		
	E-09	M	Adult	63.75	6.31	Toksun County	HE		
	E-010	M	Adult	70.99	7.92	Toksun County	HE		
	E-011	F	Adult	62.52	6.25	Toksun County	HE		
	E-012	M	Adult	66.92	6.57	Toksun County	HE		
*Phrynocephalus axillaris*	P-01	M	Adult	53.16	6.71	Toksun County	SEM, TEM	3	3, 3
	P-02	F	Adult	48.15	4.53	Toksun County	SEM, TEM		
	P-03	M	Adult	54.03	5.91	Toksun County	SEM, TEM		
	P-04	M	Adult	51.03	5.96	Toksun County	OD, HE	3	4, 2
	P-05	F	Adult	45.48	3.55	Toksun County	OD, HE		
	P-06	F	Adult	47.14	4.98	Toksun County	OD, HE		
	P-07	F	Adult	44.32	3.87	Toksun County	HE	4	2/4
	P-08	F	Adult	48.13	5.01	Toksun County	HE		
